# High-level expression of improved thermo-stable alkaline xylanase variant in *Pichia Pastoris* through codon optimization, multiple gene insertion and high-density fermentation

**DOI:** 10.1038/srep37869

**Published:** 2016-11-29

**Authors:** Yihong Lu, Cheng Fang, Qinhong Wang, Yuling Zhou, Guimin Zhang, Yanhe Ma

**Affiliations:** 1 Hubei Collaborative Innovation Center for Green Transformation of Bio-Resources, The College of Life Sciences, Hubei University, Wuhan 430062, China; 2 Tianjin institute of Industrial Biotechnology, Chinese Academy of Sciences, Tianjin, 300308, China

## Abstract

In paper industry, xylanases are used to increase the pulp properties in bleaching process as its eco-friendly nature. The xylanases activity is hindered by high temperature and alkaline conditions with high enzyme production cost in the paper industry. Here, XynHB, an alkaline stable xylanase from *Bacillus pumilus* HBP8 was mutated at N188A to XynHBN188A. Expressed mutant in *E. coli* showed 1.5-fold higher xylanase activity than XynHB at 60 °C. The mutant expressed in *Pichia pastoris* was glycosylated, remained stable for 30 min at 60 °C. XynHBN188A optimized based on codon usage bias for *P. pastoris* (*xynHBN188As*) showed an increase of 39.5% enzyme activity. The strain Y16 forming the largest hydrolysis halo in the xylan plate was used in shake flask experiments produced an enzyme activity of 6,403 U/ml. The Y16 strain had 9 copies of the recombinant *xynHBN188As* gene in the genome revealed by qPCR. The enzymatic activity increased to 48,241 U/ml in a 5 L fermentor. Supplement of 15 U/g xylanase enhanced the brightness of paper products by 2% in bleaching experiment, and thereby improved the tensile strength and burst factor by 13% and 6.5%, respectively. XynHBN188As has a great potential in paper industries.

Paper industry is one of the fastest growing sectors. The chemicals used in bleaching process are large amounts of chlorinated inorganic compounds, which are toxic, mutagenic, and carcinogenic in nature^1^. Under these circumstances, xylanase is used in the paper industry to reduce the harmful effects of chemicals causing adverse effect. The positive effect of xylanase generally attributes to the breaking down of the xylan, thereby breaking down the link between the cellulose and lignin. Once the lignin is detached from cellulose, it is readily reduced in the subsequent bleaching steps. In addition, after kraft pulping, xylan generally co-crystalize on the surface of the fiber, causing a physical barrier for the reagents to flow into the fiber^2^,^3^. Therefore, the degradation of xylan would facilitate the influx of subsequent bleaching agents. It’s known that the use of xylanase could reduce the damage to pulp fibers, and also create high quality rayon grade and superior quality dissolving pulps, providing an alternative and cost-effective method for the bleaching process^3^.

However, the high temperature and alkaline conditions influence the use of different xylanases in paper and pulp industry. Most of the presently used xylanases doesn’t withstand the high temperature or the alkaline condition causing reduced activity, which doesn’t fit to the industrial standards, so there is a great need for the thermostable xylanases effective at alkaline pH with higher activity. Xylanases were mainly classified into two families, i.e. glycosyl hydrolase GH 10 and GH 11. The GH10 families namely, endo-β-1, 4-D xylanase have a higher molecular weight (≥30 kDa), low isoelectric point, but studies have shown that these enzymes are not substrate specific. The GH11 families are the smallest xylanases (generally less than 30 kDa), which penetrate into the pulp easily with high specificity, but do not tolerate high temperature^4^. Previously, many efforts have been reported to improve the thermo-stability of xylanases by different strategies^5^,^6^. N-terminal region of xylanase was reported to play a crucial role in its thermo-stability, so single residue substitutions and chimeric alterations in the vicinity of the N-terminal region of the protein make a big difference^7^,^8^,^9^. Thermo-stability of xylanases was improved by the introduction of disulphide bridges, thereby enhancing the protein positive charges^10^,^11^,^12^,^13^,^14^. Also, increasing the stability of a protein by decreasing the configuration entropy of unfolding, such as the hydrogen bond network, can provide significant energetic contribution to the thermo-stability of a protein^15^,^16^. Deletion of the carbohydrate-binding modules (CBM) has effect on the thermo-stability of GH10 and GH11 xylanases. The truncated mutant XynATM1 harboring a GH11 catalytic module without CBM showed an improved thermal stability compared to XynA^17^. Moreover, DNA shuffling and error prone PCR were applied to engineer recombinant xylanase with improved thermo-stability^18^,^19^,^20^. These findings prompted the engineering of xylanase for large scale industrial application in paper industry, but further work is needed to develop a highly thermo-stable xylanase having better enzyme activity in alkaline condition.

The recombinant strains producing high-yield of xylanase will be better for industrial application with less downstream purification, so xylanase production will be cheaper and cost efficient^21^,^22^. Several studies have reported the role of different host strains produce high level of exogenous xylanase, like *Bacillus*, *Trichoderma reesei*, *Pichia pastoris*, *Saccharomyces cerevisiae* and *Escherichia coli*^23^,^24^,^25^,^26^,^27^. But, *P. pastoris* is known for its properties of efficient enzyme secretion, cellulase free, and fast growth with high cell density in simple media^28^. Some GH10 xylanases have been successfully expressed in *P. pastoris*, which is thermophilic but can be suitable for acidic conditions only and they showed the highest xylanase activity of 73,400 U/ml^29^.

In our previous study, the GH11 xylanase gene *xynHB* was cloned from *Bacillus pumilus* HBP8 and expressed in *P. pastoris.* The xylanase activity in the supernatant of the recombinant *P. pastoris* expressing *xynHB* was 644 U/ml^30^, and recombinant XynHB was effective in alkaline condition (pH 8.6) with 90% activity. However, the thermo-stability and expression yield did not meet the industrial standards for paper industry, since the optimal temperature of this recombinant xylanase was 40–60 °C, with only 20% residual activity after 30 min preincubation at 60 °C. In the present study, a site mutagenesis was performed to improve thermo-stability of recombinant XynHB as well as better protein expression level in *P. pastoris* by gene codon usage optimization and multiple gene insertion.

## Methods

### Strains, plasmids and chemicals

*E. coli* DH5α, *E. coli* BL21(DE3) were purchased from TransGen Biotech (Beijing, China). *P. pastoris* GS115 were purchased from Invitrogen (USA). The expression vector pET28a(+) of *E. coli* was purchased from Novagen (Germany). The recombinant plasmid pHBM130 encoding *xynHB* gene expressing xylanase^30^ and the expression vector pHBM905A of *P. pastoris*^31^ was constructed previously.

Synthesis of PCR primers and DNA sequencing were performed by GenScript Co. Ltd (Nanjing, China). Restriction enzymes, Ex Taq DNA Polymerase, LA DNA Polymerase and T_4_ DNA Ligase were purchased from TakaRa (Dalian, China). All chemicals were of analytical grade and obtained from commercial suppliers.

For culturing different recombinants of *P. pastoris*, Buffered Glycerol-complex Medium (BMGY), Buffered Methanol-complex Medium (BMMY), Minimal Dextrose Medium (MD) and basal salt medium (BSM) were prepared as described in previous study^32^.

### Construction of the *E. coli* expression plasmid and site-directed mutagenesis of *xynHB*

The target gene *xynHB* (GenBank accession number AY954630) was amplified from plasmid pHBM130 by polymerase chain reaction (PCR) using primers HB1 and HB2 (with *Bam*HI and *Sal*I site) (Table 1). Gene amplification was performed in a 50 μL PCR volume containing 10 ng of plasmid pHBM130, 0.5 μM of primer pairs, 5 μL of 10 × *Ex Taq* Buffer, 200 μM dNTPs and 1 U of *Ex Taq* DNA Polymerase (Takara). Amplification was performed in a Bio-rad T100 Thermal Cycler (USA) with 25 cycles of 94 °C for 30 s, 55 °C for 30 s, and 72 °C for 1 min. The PCR products were digested by *Bam*HI/*Sal*I and ligated into *Bam*HI/*Sal*I digested vector pET28a and transformed into *E. coli* DH5α. The recombinant plasmid pET28a-*xynHB* was confirmed by PCR and restriction enzymes digestion.

Site-directed mutagenesis was performed by the overlapping extension method using plasmid pET28a-*xynHB* as a template, and N188AF and N188AR (Table 1) as primers. PCR was performed with LA DNA Polymerase (TaKaRa, China). The purified PCR products were digested by *Dpn*I, and transformed into *E. coli* DH5α. The mutants were screened on LB plates supplemented with 50 μg/mL of kanamycin and further verified by gene sequencing.

### Expression and purification of the XynHB and XynHBN188A in *E. coli*

Recombinant plasmid was purified and transformed into *E. coli* BL21 (DE3) followed by culturing in LB-kanamycin (50 μg/ml) medium at 37 °C and 200 rpm until the OD_600_ reached 0.6. The expression of xylanase was induced with isopropylthiogalactoside (IPTG, 0.5 mM) for continuing 12 h cultivation at 18 °C. Cells were harvested by centrifugation followed by ultrasonication. His • Bind^®^ Kits (Novagen) were used according to the manufacturer’s instructions to purify xylanase. The molecular weight and homogeneity of the protein were evaluated by SDS-PAGE, followed by Coomassie Brilliant Blue G-250 staining^33^.

### Determination of xylanase activity and its thermo-stability

Xylanase were assayed by measuring the reducing sugar released from beechwood xylan by dinitrosalicylic acid method. Xylanase activity was assayed by incubating 1 mL of enzymatic extract with 1 mL of 1% beechwood xylan solution (Sigma, USA) in 50 mM Tris-HCI buffer pH 8.0 as the substrate. The mixture was incubated at 50 °C for 10 min. One unit of enzyme activity was defined as the amount of enzyme capable of releasing 1 μmol of reducing sugar from xylan per minute. Xylose was used as a standard. The protein concentration was determined by Bradford assay using bovine serum albumin (BSA) as the standard. To investigate the thermo-stability, the purified enzyme was incubated at 60 °C. Residual xylanase activity was determined at regular time intervals of 5 min in 50 °C and pH 8.0 for 10 min. The residual activity (%) was expressed as a ratio in the percentage of untreated xylanase activity. All experiments were performed in triplicate.

### The analysis of structure variation of XynHB and XynHBN188A

To investigate the structural changes responsible for the improved stability of XynHBN188A, the 3-D structure of XynHB and XynHBN188A were simulated by using MODELLER^34^. The template search for homology modeling was performed by full multiple alignments and searching in PDB. In our results, a GH11 xylanase, with its PDB number of 1IGO, was identified as the template. XynHB shared 85% amino acid sequence identity with 1IGO. Then, the structure comparison was performed by Discovery Studio (DS) client software (Accelrys Inc., San Diego, CA). The amino acids from 35 to 40 in the N-terminus were selected for the analysis of classical hydrogen bonds.

### Codon optimization of *xynHBN188A* and expression in *P. pastoris*

In order to further increase the expression level of xylanase in *P. pastoris*, the gene sequence of *xynHBN188A* was optimized based on the codon usage bias of *P. pastoris*. The codon-optimized gene was designed by DNAworks (http://helixweb.nih.gov/dnaworks/) and synthesized by Genscript (China). Primers HBsF and HBsR (Table 1) were used to amplify *xynHBN188As* gene and HB3 and HB4 primers (Table 1) for *xynHBN188A*. The partial *Cpo*I and *Not*I (TaKaRa) are underlined. The PCR products were treated with T_4_ DNA Polymerase and 1 mM dTTP for 20 min at 12 °C for overhangs. The expression vector of *P. pastoris* pHBM905A with 5′AOX1 gene promoter was digested by *Cpo*I and *Not*I restriction enzymes^35^. The treated PCR products were ligated with pHBM905A downstream of the MFα secretion signal sequence. The ligation products were transformed into *E. coli* DH5α. The transformants were screened on LB plates (100 μg/mL ampicillin), and further confirmed by colony PCR and DNA sequencing.

The recombinant plasmids (Figure S1) were linearized using *Sal*I and transformed into *P. pastoris* GS115 by electroporation (10000 V/cm, 4 ms, Bio-Rad MicroPulser Electroporator, USA). Transformants were selected on histidine-deficient MD plates and incubated at 30 °C for 3 days. Positive transformants were identified on an upside down BMMY plate containing 0.5% Remazol Brilliant Blue (RBB)-xylan with methanol dropping uniformly on the petri dish cover at 28 °C. Three colonies with the minimum halo were selected for shake flask fermentation as described in previous report with minor modification^31^. Cells were cultured in 25 ml of BMGY medium for 48 h and harvested. They were transferred into 25 ml of BMMY medium to induce xylanase expression and measured at regular intervals. Three replicates were performed for each transformant to test the activity.

### Screening of genetic-engineering yeast with high-yield XynHBN188A

Thousands of transformants were inoculated on the BMMY plates containing 0.5% (w/v) RBB-xylan, and the inducing method was described previously in the Methods 2.6. The colonies with the largest halo on each plate were inoculated on another RBB-xylan BMMY plate to perform the second level of screening to identify colonies having large halo. The colony with the largest halo was selected for shake flask fermentation.

### Determination of the copy number of *xynHBN188As* in the genome

Total DNA was isolated from *P. pastoris* according to the method of Hoffman and Winston^36^. A modified quantitative real-time PCR method was used to determine copy numbers of the target gene in cells. Glyceraldehydes-3-phosphate dehydrogenase (*GAP*) gene of *P. pastoris* was used as the reference gene. The HB3S and HB4S primers (Table 1) were used to amplify *xynHBN188As* and the GAPF and GAPR primers (Table 1) were used to amplify *GAP*. Strain P81 was used as a calibrator sample since it displayed the smallest halo on the RBB-xylan plate with minimum xylanase activity, so it is assumed that it possessed a single copy xylanase gene.

Real-time PCR (qPCR) was performed in the MiniOpticon™ Real-Time PCR System (Bio-Rad, Hercules, CA) using SYBR^®^ Premix Ex Taq™ (TliRNaseH Plus) (Takara, China). The PCR reaction mixture contained 10 ng of template DNA, 0.5 μM of primer pairs, 12.5 μL of the SYBR^®^ Premix Ex Taq™ and 25 μL sterilized distilled water. The PCR assay included an initial denaturation step at 95 °C for 5 min, followed by 40 cycles of 30 s at 95 °C, 30 s at 60 °C, and 20 s at 72 °C. Fluorescent signal measurements were carried out during the elongation step. The experiment was repeated at two independent times. The copy numbers of the *xynHBN188As* gene integrated in the genome of recombinant *P. pastoris* was calculated by the ratio of the copy numbers of the target gene against *GAP* using the ΔΔC_T_ method of relative quantification^37^.

### High-density fermentation of the recombinant yeast strain

The fermentation was carried out in a 5 L fermentor with 2 L BSM supplemented containing 8 mL/L PTM1. High cell density fermentation of *P. pastoris* was applied based on the *Pichia* Fermentation Process Guidelines (Invitrogen). The pH and the temperature were set at 6 and 28 °C, respectively. During the cell growth phase, the cells were grown until the glycerol were exhausted, which indicates the increase in dissolved oxygen (DO) level. When the glycerol was exhausted around 12 h, the feeding medium containing 50% (w/v) glycerol and 12 mL/L PTM1 solution was pumped in according to a predetermined DO level (10–20%). When the OD_600_ reached 360, the glycerol feed was stopped, which raised DO after about 30 min. At that time, pure methanol containing 12 mL/L PTM1 solution was fed to induce the targeted gene expression, and the fermentation temperature were adjusted to 25 °C, with the DO being set to 10–20%. Culture samples were taken every 12 h to determine the OD_600_, dry weight of the cells, and enzyme activity.

### Bio-bleaching of pulp

The straw pulp was provided by Wuhan Chenming Paper Co. Ltd. The usage of the xylanase is 15 U/g bone dry pulp. The usage of bleaching solution is 2%., and the enzyme to act on pulp was performed at 50 °C and pH 8.0 for 30 min. The brightness (%ISO), tensile strength and burst factor of paper were measured by YQ-Z-48A ISO Brightness color detector, D-KZW 300 Digital display tensile testing machine, and DCP-SLY1000 tensile testing machine, respectively. All these experiments were performed in triplicates.

### The accession number

The GenBank accession number of gene *xynHB* and *xynHBN188As* were AY954630 and KU188327, respectively.

### Statistical Analysis

All the experiment results were analyzed by MS Excel (MS Office 2010, USA). Differences were considered significant at *p* < 0.05.

## Results

### Improvement of thermo-stability by the mutagenesis of N188A

**Figure 1:**
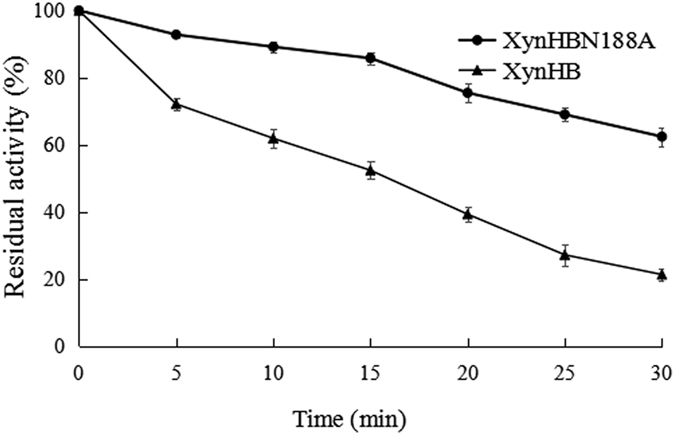
Thermo-stability of the XynHB and its mutant XynHBN188A. The thermal stability of XynHB and its mutant XynHBN188A was determined at the 60 °C in 50 mM Tris–HCl buffer (pH 8.0). After incubation, at regular time intervals of 5 min the residual activity of enzyme was measured at pH 8.0 and 50 °C. Each value of the assay was the arithmetic mean of triplicate measurements.

There are three potential glycosylation sites on the XynHB, when expressed in *Pichia,* it gets glycosylated and presented two target protein bands^30^. We had intended to study the effects of enzymatic glycosylation on three glycosylation sites in *Pichia.* When we mutated the third glycosylation site N188 to A, the result showed that the recombinant XynHBN188A in *P. pastoris* exhibited complete activity after 30 min preincubation at 60 °C, while XynHB in *P. pastoris* had only 20% residual activity after 30 min preincubation at 60 °C, indicating its enhanced endurance at high temperature. However, the extent of glycosylation of both xylanases were almost similar, consistent with the previous report in which glycosylation sites near C-terminal were not glycosylated or less glycosylated^38^. So, we predicted the improvement of thermo-stability maybe not related to its glycosylation. In order to determine whether the site mutation caused an increase in thermal stability, we expressed the site mutated protein XynHBN188A in *E. coli.* Recombinant xylanases were purified and evaluated according to the methods 2.3 (Figure S2). A 26 kDa band was observed, corresponding to the predicted size, 3 kDa larger than the native xylanase (23 kDa), since recombinant xylanase have 34 amino acids more in N-terminal^30^. The thermo-stability of purified xylanase was evaluated by measuring its residual activities after incubation at 60 °C at different time intervals (Fig. 1). Results indicated that the thermo-stability of the XynHBN188A was 1.5-fold higher than that of XynHB at 60 °C for 30 min. The other characters of XynHBN188A like the optimum temperature (50 °C), pH (8), pH stability, specific activity (2,510 mg/mL) were identified, but no significant difference with the wild-type XynHB.

**Figure 2:**
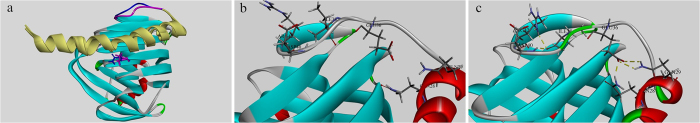
Homology modeling and structure comparison of wild type XynHB and XynHBN188A. (**a**) The predicted modeling structure of XynHB and XynHBN188A. Dark blue line is the structure of the wild type XynHB while purple is the mutant enzyme XynHBN188A. The mutation sites were shown by ball and line. The 34 amino acid sequence in the N-terminal which were from the vector was marked in yellow. (**b**) The N-terminus structure of wild type XynHB. (**c**) The N-terminus structure of mutant XynHBN188A.

To evaluate the effects of mutation N188A on the improvement of xylanase’s thermo-stability, the structures of XynHB and XynHBN188A were modeled by homology modeling and further analyzed using DS program. Interestingly, the overall three-dimensional structures of XynHB and XynHBN188A are practically identical except the N-terminal region (Fig. 2a). The detailed N-terminus structure of XynHB and XynHBN188A had two major differences as shown in Fig. 2b and c. One difference is that the two hydrogen bonds formed between Arg42 and Ile38, Asp40 were only observed in XynHBN188A, dragging the N-terminus to the middle core structure of the protein. The other is Glu36 formed three new hydrogen with Glu28, Glu29 only in XynHBN188A, strengthening the electrostatic interactions between the amino acids in N-terminal region of the mutant. The mutant expressed in *Pichia* doesn’t have more 34 amino acids from the vector and its thermo-stability also improved, so we assumed the two hydrogen bonds formed between Arg42 and Ile38, Asp40 might have helped to form an intense structure, responsible for the improved thermo-stability of the mutant XynHBN188A.

### Codon optimization enhance the expression of xylanase

**Figure 3:**
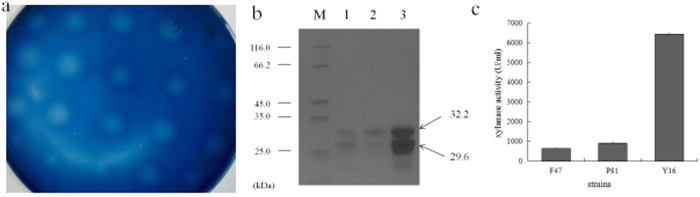
(**a)** Screening of the high-copy number strain Y16. (**b**) SDS-PAGE analysis of high-yield strain Y16. M, Protein molecular weight standard. 1, Crude enzyme expressed by P81 strain. 2, Crude enzyme diluted 10 times expressed by Y16 strain. 3, Crude enzyme expressed by Y16. (**c**) Codon usage and high copy number improves the expression yield of XynHBN188A in *P. pastoris*.

In order to further increase the expression level of xylanase in *P. pastoris*, the gene sequence of *xynHBN188A* was optimized based on codon bias of *P. pastoris*. Total 139 nucleotides were substituted in the optimized sequence of *xynHBN188As*. The G + C content of the *xynHBN188As* was 39.60% compared to 42.41% in *xynHBN188A*. The recombinant strains presenting the smallest halo in the RBB-Xylan plate were picked up as described in the Methods 2.6, and the isolated strains containing *xynHBN188As* and *xynHBN188A* were named as P81 and F47, respectively. P81 exhibited a xylanase activity of 907 U/ml in the culture supernatant, while F47 had a xylanase activity of 650 U/ml. This result suggested that enzyme activity enhanced by codon optimization was 39.5% (Fig. 3c).

### Screening of recombinant *Pichia* with high expression of xylanase

The *xynHBN188As* were integrated into the genome of *P. pastoris* GS115 and grown on MD plate. Single colonies were re-inoculated on the BMMY plate containing 0.5% RBB-xylan, followed by induction with methanol. The colony forming the largest hydrolysis halo on the plate indicates the highest xylanase activity with more gene copies integrated in the *P. pastoris* genome, and the identified colony in our study was named as Y16 and set for further evaluation (Fig. 3a).

Further biochemical characterization of high-copy strain Y16 and single-copy strain P81 were performed. The culture supernatants of Y16 and P81 in shaking flask fermentation were detected via SDS-PAGE (Fig. 3b), in which two protein bands with molecular weights of approximate 29.6 kDa and 32.2 kDa were observed. In our previous published study^30^, we have performed the zymography analysis to confirm that the two protein bands (32.2 kDa and 29.6 kDa) were recombinant xylanase, and EndoH treatment to testify that they were both glycosylated products, and the native xylanase from *B. pumilus* HBP8 was used as a control. The protein yield of the Y16 strain was around 10 times higher than that of P81 (Fig. 3b). Xylanase activity in the supernatant of Y16 was 6,403 U/ml, while P81 was 907 U/ml (Fig. 3c).

The high copy number of integrated gene in the host chromosome normally led to the higher expression yield of target proteins. Therefore, qPCR was performed to determine the copy number of the exogenous *xynHBN188As* gene in the genome of Y16. P81 was used as a calibrator strain, and *GAP* gene of *P. pastoris* was used as the reference gene. The qPCR analysis was performed according to the Methods 2.8. The mean *C*_T_ values for the target and internal control genes were calculated and the fold difference was determined as 2^ΔΔ*C*T^, which was calculated as the equation below, ΔΔC_T_ = (C_T_,_*xynHBS*_ − C_T_,_*GAP*_)_Test_ − (C_T_,_*xynHBS*_ − C_T_,_GAP_)_Calibrator_^28^. The results (Table 2) indicated that the copy number of *xynHBN188As* in the Y16 strain was 9.15.

### High-density production of xylanase in *Pichia* strain Y16

**Figure 4:**
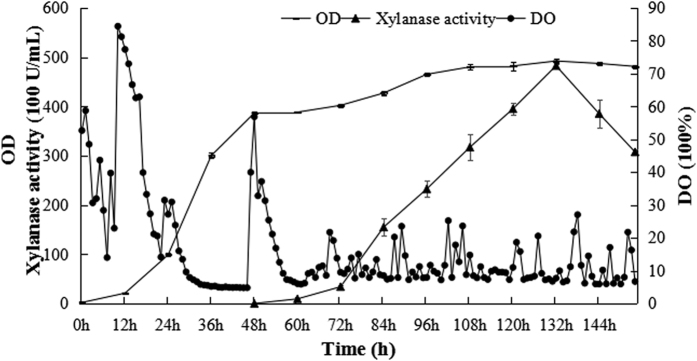
DO, OD and xylanase activity curves in a 5 L fermentor.

**Figure 5:**
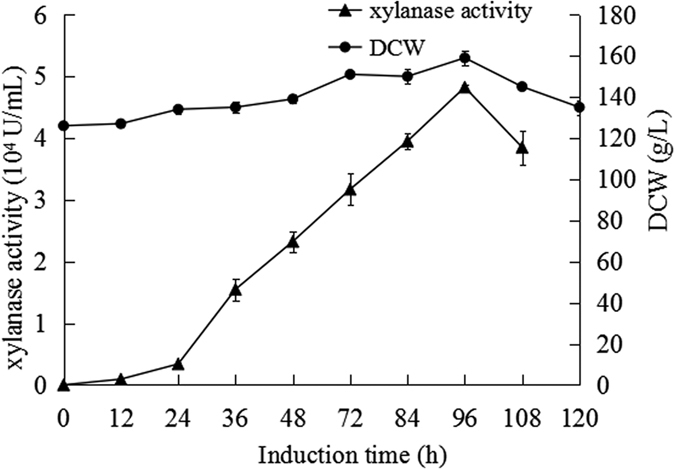
Xylanase activity and cell dry weight of Y16 in a 5 L fermentor.

Fed-batch fermentation of the strain Y16 was carried out in a 5 L fermentor by using a three phase growth protocol (Fig. 4). The glycerol batch phase was first employed for 12 h after inoculation. The glycerol fed-batch phase terminated after 35 h when the dry cells weight (DCW) reached 126 g/L. When the glycerol feed was stopped, the DO increased approximately 60%. In the third phase, the methanol fed-batch phase was started to induce the production of xylanase by feeding methanol in a stepwise increasing rate. No apparent xylanase activity was detected until the methanol fed-batch phase was initiated. After the methanol fed-batch phased for 96 h, the cell density reached 159 g/L, and the maximum xylanase activity in the supernatant was 48,241 U/mL (Figs 4 and 5). This enzyme activity was 7.5 times higher than that of the shake flask fermentation, which was only 6,403 U/ml.

### Bio-bleaching studies using the straw pulp

The application of XynHBN188A on the straw pulp was also analyzed by adding the fermentation broth of Y16 onto the straw pulp. The results (Table 3) indicated that the paper treated with recombinant xylanase exhibited better quality in brightness, tensile strength and burst factor compared to the control without xylanase addition. The supplement of 15 U/g xylanase enhanced the brightness (%ISO) by 2%, the tensile strength by 13% and the burst factor by 6.5%.

## Discussion

The chemicals used for paper bleaching process resulted in the release of large amounts of chlorinated organic compounds that are known to have toxic, mutagenic and carcinogenic effects, causing serious environmental pollution. After being subjected to chemical bleaching, xylanase treated pulp results in reduction of chlorine and chlorine dioxide consumption, along with a reduction in COD value with significant improvement in various pulp properties. Therefore, developing a cost-efficient bleaching process with xylanase represents a positive benefit to paper industry, in which the low-cost alkaline and thermo-stable xylanase is a key issue.

In our earlier study, xylanase was effective in alkaline condition (pH 8.6) with 90% activity and its activity in the supernatant of the recombinant *P. pastoris* expressing *xynHB* was 644 U/ml^30^. However, such thermo-stability and enzyme expression yield did not meet the industrial demand of paper production. The thermo-stability of XynHB was first improved. Previous reports indicated that N-terminal region and hydrogen bond network of xylanase played crucial roles in its thermo-stability^8^,^9^,^15^. GH11 xylanases of thermophilic origin generally have longer N-terminal region compared to mesophilic ones, indicating its role on enhancing the thermo-stability^39^,^40^. Several attempts have been made to enhance the thermo-stability of xylanase by substituting the N-terminal region of various non-thermostable xylanases with those of thermostable xylanases^8^. We first mutated N188 site which led to the increase in the thermo-stability of xylanase XynHB, and its thermo-stability tends to be higher and effective at 60 °C. From the homology modeling analysis between the structures of the wide type and mutant xylanase, there was difference in the N-terminus. The N-terminus of the mutant became more nearer to the middle core structure of the protein, which results in a more intense overall structure. This was similar to the thermo-stability of xylanase from *Aspergillus niger* and mesophilic xylanase SoxB from *S. olivaceovirdis* was largely enhanced by substituting their corresponding N-terminal regions with xylanase A (TfxA) from *T. fusca*^7^. Furthermore, hydrogen bond network, as one of the most important interactions for maintaining the protein’s secondary structures, can provide significant energetic contribution to the thermo-stability of a protein^15^,^16^. Previous studies have indicated that hydrogen bonds were the key to the main differences between *B. circulans* xylanase and the thermophilic *T. lanuginosus* xylanase^41^, the two hydrogen bonds formed between Arg42 and Ile38, Asp40 were only observed in XynHBN188A, dragging the N-terminus to the middle core structure of the protein, which in-turn strengthened the electrostatic interactions between the amino acids in N-terminal region of the mutant. These major structural differences might have helped to form an intense structure, responsible for the improved thermo-stability of the mutant XynHBN188A.

*P. pastoris* is able to perform many eukaryotic post-translational modifications, including proteolytic processing, disulfide bond formation and glycosylation. Moreover, it can also efficiently secrete heterologous protein with low level of background protein, which makes it for zero need in disrupting the cells, simplifying the purification process of the secreted heterologous protein for paper making. More importantly, *P. pastoris* is specifically attractive for enzymes used in paper industry because it does not produce endogenous cellulases that weaken the fiber. Thus, *P. pastoris* is an ideal host for xylanase expression. In the present study, an optimized *xynHBN188As* was over expressed in *P. pastoris* by codon optimization and functional screening, with more than nine copies of xylanase gene on the genome of *P. pastoris*. In the previous studies, the strains containing high gene copies with high protein yield were screened by antibiotics resistance, such as G418 and zeocin. The constructed strains made by this method contained several antibiotic resistance genes, increasing the risk of biological safety. But the constructed strain Y16 generated in this study did not contain any antibiotic resistance gene, avoiding the potential biosafety issues.

Till date, the reported highest xylanase activity was 73,400 U/ml in 3.7 L fermentor, which was obtained by an acidic xylanase, from the acidophilic fungus *Bispora* sp. MEY-1^29^. The xylanase activity in our recombinant stain reached 48,241 U/mL in 5 L fermentor after 96 h induction, showing the highest xylanase activity at alkaline condition compared to the earlier published reports. The xylanase activity in our recombinant stain reached 48,241 U/mL which is much higher than the xylanase activity in the supernatant of the recombinant *P. pastoris* expressing *xynHB* was 644 U/ml from our earlier study before modification^30^. Structural modification of the protein and the alteration in the fermentation increased the xylanase activity to a larger extent, which could be used in the industrial level as the enzyme being highly stable at pH 7.5 to 8.6 and 50 °C with maximum activity. Further application results in the pulp bleaching process indicated that the recombinant xylanase exhibited better effects in improving the quality of paper.

In summary, due to its alkaline, thermostable properties with high-yield in *P. pastoris,* the mutant XynHBN188A is a potentially good candidate for industrial application and crude enzyme solution of Y16 strain is good to be used directly in bleaching pulp without purification. In fact, the subsequent pulp bleaching experiments provided direct evidence that crude enzyme solution could improve the qualities of the paper in brightness, tensile strength and burst factor.

## Additional Information

**How to cite this article**: Lu, Y. *et al*. High-level expression of improved thermo-stable alkaline xylanase variant in *Pichia Pastoris* through codon optimization, multiple gene insertion and high-density fermentation. *Sci. Rep.* **6**, 37869; doi: 10.1038/srep37869 (2016).

**Publisher's note:** Springer Nature remains neutral with regard to jurisdictional claims in published maps and institutional affiliations.

## Supplementary Material

Supplementary Information

## Figures and Tables

**Table 1:** The primers used in this study.

Primers	Sequence
HB1	5′-CGCGGATCCGCGGAAACGATTTATGATAATAGA 3′
HB2	5′-ACGCGTCGACCTTTTATCGAATCATCAGCTGA 3′
N188AF	5′-AGAAGGCTACCGAAGCGCCGGAAG 3′
N188AR	5′-CATTCGCACTTCCGGCGCTTCGGT 3′
HBsF	5′-GTCAGCGGAAACGATTTATGATAATAGA 3′
HBsR	5′-GGCCACTTTTATCGAATCATCAGCTGA 3′
HB3	5′-GTCAGCGGAAACGATTTATGATAATAGAATAGG 3′
HB4	5′-GGCCATTATCGAATCATCAGCTGATTCGTCAT 3′
GAPF	5′-GGTATTAACGGTTTCGGACGTATTG-3′
GAPR	5′-GATGTTGACAGGGTCTCTCTCTTGG-3′
HB3S	5′-TATTGTTGAGTCCTGGGGAACTTAT-3′
HB4S	5′-AACAGACCAATATTGCTTAAAAGT-3′

**Table 2:** Calculation of the copy number of *xynHBN188As* in the genome of Y16 strain.

Strain	Gene	Description	*C*_T_ value	Mean *C*_T_ value	2^−ΔΔ*C*T^	The copy number of *xynHBN188As*
P81	*GAP*	Ref; Cal	19.19	19.04		
P81	*GAP*	Ref; Cal	18.77			
P81	*GAP*	Ref; Cal	19.16			
Y16	*GAP*	Ref; Test	19.07	18.97		
Y16	*GAP*	Ref; Test	18.86			
Y16	*GAP*	Ref; Test	18.97			
P81	*xynHBs*	Target; Cal	19.12	18.95	0.89	1
P81	*xynHBs*	Target; Cal	18.96		0.99	
P81	*xynHBs*	Target; Cal	18.78		1.12	
Y16	*xynHBs*	Target; Test	15.70	15.69	9.06	9.15
Y16	*xynHBs*	Target; Test	15.69		9.13	
Y16	*xynHBs*	Target; Test	15.67		9.25	

Ref represent reference gene *GAP*; Cal represent the single copy stain P81; Target represent target gene *xynHBN188As*; Test represent Y16 strain.

**Table 3:** Bio-bleaching of XynHBN188A on the straw pulp.

	Without the enyzme	Fermentation broth of Y16
Brightness (%ISO)	82.09 ± 0.12	84.12 ± 0.21
Tensile strength (N × m/g)	15.76 ± 0.06	17.81 ± 0.08
Yield (%)	86.5 ± 0.21	84 ± 0.25
Burst factor (kPa × m^2^/g)	0.91 ± 0.02	0.97 ± 0.01
