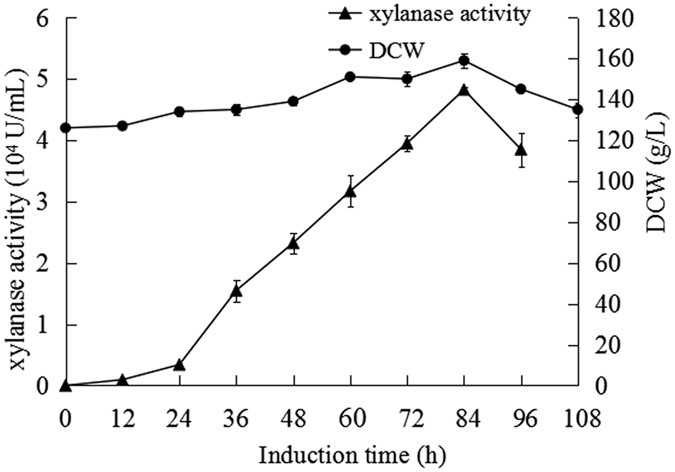# Corrigendum: High-level expression of improved thermo-stable alkaline xylanase variant in *Pichia Pastoris* through codon optimization, multiple gene insertion and high-density fermentation

**DOI:** 10.1038/srep44719

**Published:** 2017-03-16

**Authors:** Yihong Lu, Cheng Fang, Qinhong Wang, Yuling Zhou, Guimin Zhang, Yanhe Ma

Scientific Reports 6: Article number: 3786910.1038/srep37869; published online: 11 29 2016; updated: 03 16 2017

This Article contains an error in the x-axis labelling of Figure 5. The correct Figure 5 appears below as Figure 1.

## Figures and Tables

**Figure 1:**