# Supplementary material for: High-level expression of improved thermo-stable alkaline xylanase variant in Pichia Pastoris through codon optimization, multiple gene insertion and high-density fermentation
Source: Sci Rep. 2016 Nov 29;6:37869. doi: 10.1038/srep37869 (PMC5126662; doi:10.1038/srep37869)

**High-level expression of improved thermo-stable alkaline xylanase variant in *Pichia Pastoris* through codon optimization, muliple gene insertion and high-density fermentation**

Yihong Lu^1*^, Cheng Fang ^1*^, Qinhong Wang^2^, Yuling Zhou^1^, Guimin Zhang^1#^, Yanhe Ma^2^

Affliations:

^1^Hubei Collaborative Innovation Center for Green Transformation of Bio-Resources, The College of Life Sciences, Hubei University, Wuhan 430062, China

^2^Tianjin institute of Industrial Biotechnology, Chinese Academy of Sciences, Tianjin, 300308, China

*, Contribute equally

#, Correspondence: Zhang Guimin, The College of Life Sciences, Hubei University, Wuhan 430062, China; Tel: +86 27 88661746-8026; Fax: +86 27 88666081; E-mail: [zhangguimin6@hotmail.com](mailto:zhangguimin6@hotmail.com)

Yihong Lu, daystarlu@gmail.com

Cheng Fang, playfang1989@gmail.com

Qinhong Wang, wang_qh@tib.cas.cn

Yuling Zhou, zyl609@sina.com

Guimin Zhang, zhangguimin6@hotmail.com

Yanhe Ma, [mayanhe@im.ac.cn](mailto:mayanhe@im.ac.cn)

**Figure S1 The map of xylanase gene expression plasmid in *Pichia pastoris*.**


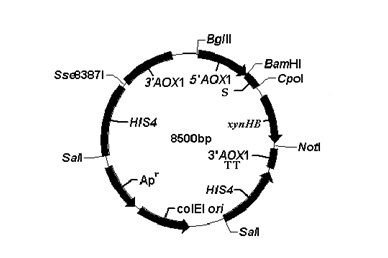


**Figure S2 SDS-PAGE analysis of recombinant xylanase in *E. coli*.** M, Protein molecular weight standard. 1, XynHB. 2, The mutant XynHBN188A.


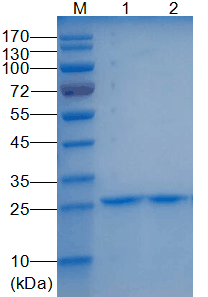

Supplement: Supplementary Information [file srep37869-s1.doc]
